# Telerehabilitation acceptance among patients during Circuit Breaker period: A retrospective study

**DOI:** 10.1016/j.dialog.2022.100049

**Published:** 2022-09-24

**Authors:** Boo Keong Fang, Jaclyn Jieying Jiang, Jonathan Kok Seng Loh, Shiek Abdullah Bin Ismail

**Affiliations:** Yishun Health, Department of Rehabilitation Services, Singapore

**Keywords:** Health services, Telerehabilitation, Rehabilitation, Outpatient

## Abstract

**Background and objectives:**

During the COVID-19 pandemic, telerehabilitation (TR) was viewed as an ideal alternative to minimize infection risks with in-person rehabilitation. However, TR acceptability remains unclear as it is an unfamiliar mode of interaction for most patients. We aim to retrospectively: (i) review the uptake rate of TR among patients, and (ii) explore their perceived barriers and facilitators of the service.

**Design:**

A cross-sectional retrospective study was conducted among eligible patients who were offered TR. Research data was extracted from medical records. Additionally, an anonymous patient satisfaction survey was conducted among the successfully enrolled patients, and the feedback was extracted from the form.gov.sg server.

**Result(s):**

24·2% of the 314 eligible patients were successfully enrolled into TR. Preference for in-person rehabilitation was the top reason cited for declining the service. Among the 157 patients who declined the service, 38·2% of them preferred in-person rehabilitation over TR.

**Conclusion(s):**

A low uptake of TR services was demonstrated, with preference for in-person rehabilitation being the majority cited reason for decline. Reconciling the differences in patients' perceptions between in-person rehabilitation and TR may improve uptake rates.

## 1 Introduction

In light of the COVID-19 global pandemic, the national taskforce of Singapore implemented Circuit Breaker (CB) as a mitigative measure from 7 April 2020 [1] to 1 June 2020 [2], to curb the spread of the disease. During the CB period, close-contact services, including conventional (in-person) physiotherapy, occupational therapy and speech therapy, were suspended or kept to a minimum. It consequentially gave rise to a new access barrier to these services, additional to known barriers in the uptake of outpatient rehabilitation, which includes (i) financial burden, (ii) inconveniences of transportation, (iii) infrastructure limitation, and (4) lack of awareness and knowledge of the benefits of rehabilitation [[3], [4], [5], [6]].

As conventional rehabilitation was kept to a minimum, care opportunities were either deferred or delayed. Rehabilitation is recommended for post-surgical recovery, individuals suffering from a recent cerebrovascular injury, or for those who require structured programs to attenuate frailty and reduce fall risks [7]. Deferral or delay of rehabilitation care for these individuals can have significant consequences, such as increased joint stiffness which may limit mobility and quality of life [8,9], increase frailty and risk of falls [10], and increase risk of hospital readmission and mortality rate in some cases [11]. To mitigate the adverse effects of these barriers to outpatient rehabilitation, telerehabilitation (TR) was seen as an ideal alternative approach to access rehabilitation care during this period [9,[12], [13], [14]].

Telerehabilitation refers to the use of virtual platforms to render rehabilitative services to people remotely in their home or other environments [15,16]. The aspects of patient care within TR includes, patient consultation and interview, assessment and diagnosis, treatment, education, and training to maintain wellbeing [17]. Telerehabilitation has also been shown to be a feasible and effective alternative to conventional rehabilitation on motor, cortical and mood disorder post stroke [18], cardiac rehabilitation [[19], [20], [21]], musculoskeletal conditions [12], and post total knee arthroplasty rehabilitation [22].

While provision of TR for patients needing outpatient rehabilitation services is an attractive proposition, it remains a relatively new mode of interaction for most patients. This is because conventional outpatient rehabilitation services have been the common modus operandi in Singapore prior to the onset of the pandemic [23]. Additionally, outpatient rehabilitation services in Singapore are geographically accessible [24].

It is thus unknown as to how TR will appeal to Singaporeans. This paper aims to scope TR acceptance by retrospective (i) review of the uptake rate amongst existing patients requiring outpatient rehabilitation service in service in Yishun Health: consisting of Khoo Teck Puat Hospital, Yishun Community Hospital, and Admiralty Medical Centre; and (ii) explore their perceived barriers and benefits of the service.

## 2 Methods

### 2.1 Design

This cross-sectional retrospective study was approved by the National Health Group (NHG) Domain Specific Review Board (DSRB) (NHG DSRB reference: 2020/00918).

### 2.2 Participants

All patients who were receiving outpatient rehabilitation, inclusive of physiotherapy, occupational therapy and speech therapy services at Yishun Health, were first screened by the respective outpatient therapists to determine eligibility for inclusion into TR. The therapists would justify the eligibility based on the information in their medical record, which was based on a pre-determined inclusion/exclusion criterion, created by a panel of therapists, with a minimum of four years of experience, working at the respective clinics (Appendix A).

**Fig. 1:**
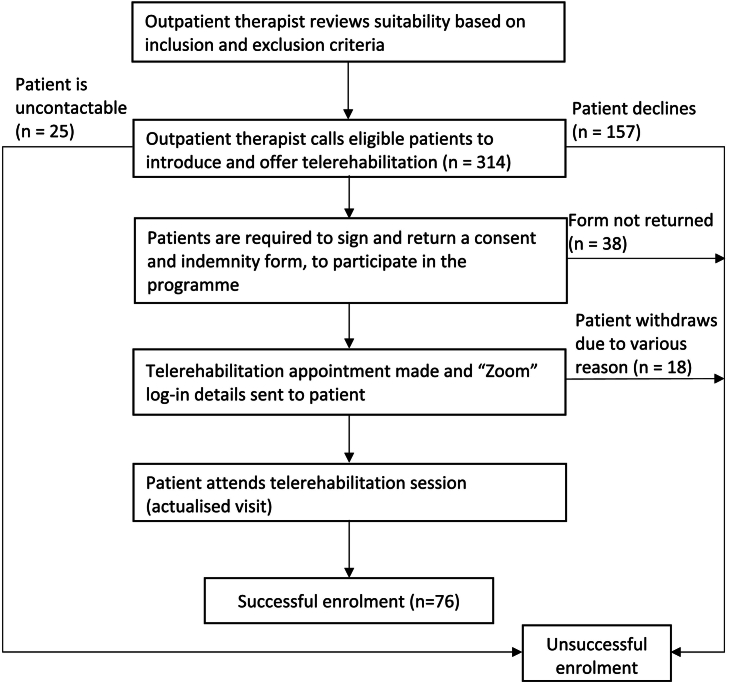
Flow chart on enrolment of eligible patients.

Eligible patients were then invited to enroll in the TR service and were required to return their signed consent and indemnity forms to formalize their interest to participate. They were also provided a handout, in either English or Mandarin, detailing instructions for the download and use of the Zoom application. Guidelines were also provided to optimize the TR session experience. There were no specific instructions on preferred devices to be used. Successful enrolment is defined as having an actualized TR visit (Fig. 1). Patients who declined the service were invited to share their reasons through phone interviews. A follow up phone call enquiry was also made to the patients who withdrew from the service. The communication between patients and therapists were carried out through phone communication, electronic mail, or the TigerConnect (secured messaging) application. The data was extracted retrospectively from electronic medical record and it was not identifiable, therefore DSRB had approved the waiver of informed consent.

### 2.3 Intervention

Telerehabilitation was conducted by therapists with a minimum of four years of experience. The therapists received training on safety and guidelines with provision of TR services, via electronic platform Licensing Experimentation and Adaptation Program (LEAP), which is a Ministry of Health sandbox initiative to support the development of telemedicine services [25].

“Zoom” (Zoom Video Communications, San Jose, CA, USA) was the video communication platform chosen for delivery of TR. The therapists underwent training on the use of the application within their specific areas. Therapists were also equipped with an internet-enabled corporate laptop, high-definition web camera, and either a television or projector for better visualization of the session.

At the beginning of the session, each therapist would conduct subjective and objective assessment, and prescribe interventions based on clinical findings. The interventions may consist of flexibility, strengthening and functional exercises, speech and swallowing training, patient education, or advice for activity modifications. Each session was planned to last for 30 to 45 min and the patients were provided with a patient experience survey at the end of the session.

### 2.4 Data collection

The data extraction process was performed in a single sitting by the research team, after receiving approval from the NHG DSRB (NHG DSRB reference: 2020/00918), and the hospital Medical Records Office (DE-2074,16-09-2020).

Data was retrospectively collected from medical records of all patients who were referred to outpatient rehabilitation at Yishun Health from 7th April 2020 to 18th June 2020. Our data gathering includes patients, referred to physiotherapy, occupational therapy and speech therapy, who were eligible for TR. Clinical research data was extracted from electronic platform, Sunrise Clinical Manager, or paper case files.

The patient experience survey was created by the institution for the purpose of collecting patients' feedback on their satisfaction with the service. This survey was designed to obtain feedback on patients’' experience and perception towards TR sessions, using 5-point Likert scale and a multi-select multiple choice question for their opinion on how TR helped them, as shown in Table 4. Survey data collected was extracted subsequently from the encrypted server form.gov.sg.

### 2.5 Data analysis

Data was analyzed using IBM SPSS Statistics version 27. Continuous variable such as age was presented as mean and standard deviation (SD). Discrete variables such as gender, disease classification in accordance to the Australasian Rehabilitation Outcomes Centre (AROC) impairment codes [26], and ethnic distribution, were presented in percentages. Descriptive analysis was performed to summarize the baseline characteristics of all participants.

Thematic analysis was applied for the reasons cited for declining TR [27]. The investigators independently reviewed the texts to familiarize and identify key phrases or concepts within the content. Coding of key phrases and concepts were performed, and codes were grouped to identify themes as shown in Table 3. The investigators then underwent a consensus exercise together to agree on the themes that were identified and verify that it is representative of the responses. Differences in opinion were raised and put to majority voting. Datasets with missing demographic information and surveys with missing responses were excluded from the analysis.

### 2.6 Role of the funding source

This research received no specific grant from any funding agency in the public, commercial or not-for-profit sectors.

## 3 Results

### 3.1 Demographics

During CB period, 314 patients were offered TR. The mean age was 49 years and 57·0% (*n* = 179) of them were male. Of those 314 patients, 62·1% (*n* = 195) of them were Chinese, 24·2% (*n* = 76) were Malay, 8·9% (*n* = 28) were Indian and the remaining 4·8% were from other ethnic groups. 43·3% (*n* = 136) of them reported employment and majority of them, 86·9% (*n* = 273), were staying in public housing. 96·2% (*n* = 302) of the patients had prior experience of in-person rehabilitation. Clinical diagnoses of these patients were categorized into 12 themes are shown in Table 1.

**Table 1:** Demographic.

Variables	Percentages
Total participants	314
	
Source	
• Day Rehabilitation Centre (Physiotherapy & Occupational Therapy)	28.4%
• Outpatient Physiotherapy	64.0%
• Outpatient Speech Therapy	7.6%
	
Age	Mean (SD)
	49 ± 21
	
Gender	
• Females	42.9%
• Males	57.0%
	
Ethnicity	
• Chinese	62.1%
• Malay	24.2%
• Indian	8.9%
• Others	4.8%
	
Diagnoses	
• Amputation	3.8%
• Brain dysfunction	0.6%
• Cancer	1.9%
• Cardiac	0.6%
• Deconditioning	7.6%
• Developmental disabilities	0.3%
• Hip fracture	4.1%
• Musculoskeletal conditions	59.9%
• Neurological conditions	4.8%
• Spinal cord injury	0.3%
• Stroke	14.0%
• Other	1.9%

### 3.2 Participation

**Fig. 2:**
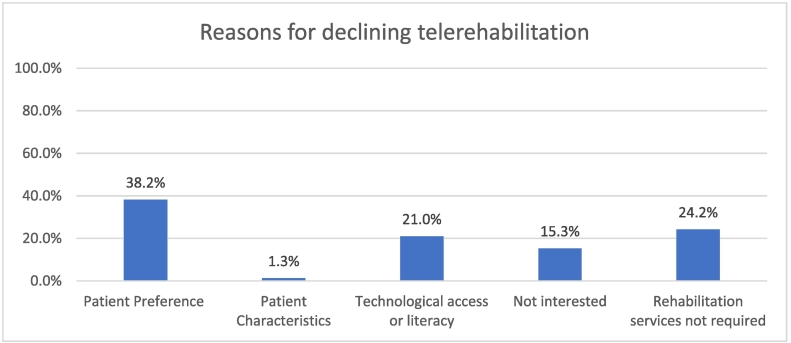
Distribution of patients who declined telerehabilitation.

Out of 314 patients, 7·9% (*n* = 25) of them were not contactable and half (*n* = 157) declined the offer of TR, as shown in Table 2. Out of 157 patients who declined the service, 38·2% (*n* = 60) of them preferred in-person sessions over virtual ones and 15·3% (*n* = 24/157) of them expressed lack of interest in TR. Another 21·0% (*n* = 33) declined the service due to lack of technological hardware, access, or literacy. Nearly a quarter of the patients, 24·2% (*n* = 38) did not require follow up services as they were either keen for self-management, have made improvements, returned to work, or are currently seeking rehabilitation elsewhere and 1·3% (n = 2) of them were found to be not suited for TR when contacted, as shown in Fig. 2.

**Table 2:** Participation.

Variables	Percentages
Total Participants	314
Agreeable	42·0%
Decline	50·0%
Uncontactable	7·9%
Successful enrolment:	
• Actualized o Male o Female	24·2% 69.7% 30.3%
Reasons for withdrawal (*n* = 18)	
• Busy working	11·1%
• Missed appointment	5·6%
• No appointment given	5·6%
• Preferred face to face session	11·1%
• Session scheduled post Circuit Breaker	50·0%
• Withdrew	16·7%

Table 2 also showed that 132 patients were agreeable with the offer and 57·6% (*n* = 76) of them were successfully enrolled into the program, reflecting a 24·2% (n = 76 out of 314) uptake rate. Out of 76 patients who enrolled to the service, 69.7% (*n* = 53) of them were male and the rest were female. Of the remaining 42·4% (*n* = 56) who were not successfully enrolled, 67·9% (*n* = 38) of patients did not return their consent form, as shown in Fig. 1. The remaining 18 patients were withdrawn for various reasons. Half of the patients (*n* = 9) had their TR session scheduled after phase 1 of CB measure, which was out of the study period. Another 16·7% (n = 3) of them withdrew from the services with no clear reason provided. 11·1% (*n* = 2) of patients still preferred face-to-face session and another 11.1% (n = 2) dropped out due to work commitments. Lastly, 11·1% (n = 2) of them missed their appointment or had no appointment given to them.

### 3.3 Survey responses

Table 4 showed that 18·9% (*n* = 25) of successfully enrolled patients responded to the patient experience survey. Most of the respondents agreed that the Zoom application is easy to use, they were able to view the therapist and materials online clearly, they were able to hear the therapist online clearly, and they were satisfied with the TR session, reporting 92·0% (*n* = 23), 92·0% (n = 23), 92·0% (n = 23) and 84·0% (*n* = 21) respectively, as shown in Table 4. Table 4 also showed that 48·0% (*n* = 12) of the respondents agreed with the statement that the TR session was equivalent to a physical session whilst 40·0% (*n* = 10) disagreed. Table 3 showed that slightly less than half of them, reporting 48·0% (*n* = 12), were happy to attend another virtual session, 36·0% (*n* = 9) remained neutral and 16·0% (*n* = 4) disagreed with it.

**Table 3:** Themes for declining telerehabilitation.

Patient Preference	• Prefer face to face or physical session • Prefers face to face session as speech therapist able to provide auscultation and palpation • Prefers to resume rehabilitation when Circuit Breaker ends
Patient Characteristics	• Advance dementia • Does not interact well through video interface
Technological Access or Literacy	• Using basic phone or no smart devices • Lack technological knowledge • No WIFI available • Does not have device with camera • Not technology savvy
Not Interested	• Wants to consider • Declined service despite persisting symptoms • Uncomfortable or unfamiliar with video call platform
Rehabilitation Services not Required	• Keen for self-management or able to manage • Coping well or no more complaints or issues • Improving or improvement • Feels better • Returned to work or army camp • Followed up with private setting physiotherapist instead • Readmission to acute hospital

Table 3 also showed that 60·0% (*n* = 15) agreed that TR helped continue their rehabilitation in the multiple selection question. Approximately half of them agreed that it allowed them to have access to a therapist, to clear any doubt they had about their condition, and to clear any question they had about their prescribed exercise, reporting 48·0% (n = 12), 52·0% (*n* = 13) and 52·0% (n = 13) respectively, as shown in Table 4.

**Table 4:** Survey responses.

**Response**	**Percentages**
Total Respondents	25
I found the ZOOM application easy to use:	
• Strongly Agree	48·0%
• Agree	44·0%
• Neutral	0·0%
• Disagree	8·0%
• Strongly disagree	0·0%
I was able to see the therapist and materials online clearly:	
• Strongly Agree	60·0%
• Agree	32·0%
• Neutral	8·0%
• Disagree	0·0%
• Strongly disagree	0·0%
I was able to hear the therapist online clearly:	
• Strongly Agree	56·0%
• Agree	36·0%
• Neutral	4·0%
• Disagree	4·0%
I am satisfied with the telerehabilitation session:	
• Strongly Agree	48·0%
• Agree	36·0%
• Neutral	4·0%
• Disagree	4·0%
• Strongly Disagree	8·0%
I found the telerehabilitation session equivalent to a face-to-face session:	
• Strongly Agree	4%
• Agree	44%
• Neutral	12%
• Disagree	20%
• Strongly Disagree	20%
I will be happy to attend another telerehabilitation session in the future:	
• Strongly Agree	16·0%
• Agree	32·0%
• Neutral	36·0%
• Disagree	4·0%
• Strongly Disagree	12·0%
I found telerehabilitation has helped me to:	
• Continue with my rehabilitation	60·0%
• Allow me to have access to a therapist	48·0%
• Allow me to clear any doubts I have about my condition	52·0%
• Allow me to clear any question I have about my prescribed exercises	52·0%

## 4 Discussion

Retrospectively, our study demonstrated a low (24·2%) uptake of TR services by patients who have been receiving outpatient care at Yishun Health during the CB period. Continuity of care (60·0%), and access to a therapist (48·0%) were the key benefits identified which motivated the patients to take up this service.

The benefits identified by our study supports the adoption of TR as a necessary adaptation to ensure continued access to rehabilitation during the pandemic [28]. Similarly, in the post-COVID era, TR may facilitate care for some patients who may face other barriers in attending conventional outpatient rehabilitation [29]. In addition, studies have shown that TR does not increase risk of adverse event compared to conventional rehabilitation [9,[30], [31], [32], [33], [34]]. Apart from the above identified benefits, the study's inclusion of patients with a wide spectrum of conditions, highlighted the ability of delivering TR services for patients different conditions [8,35]. This is in line with a recent study that summarized the findings of 53 systematic reviews. This study concluded that telerehabilitation in physiotherapy could be comparable with in-person rehabilitation or better than no rehabilitation for conditions such as osteoarthritis, low-back pain, hip and knee replacement, and multiple sclerosis and also in the context of cardiac and pulmonary rehabilitation [16].

Contrastingly, preference for in-person sessions (38·2%) was the top reason cited for declining TR and another one-sixth of patients (15·3%) expressed a lack of interest. The patient's preference was also re-iterated in the reasons for withdrawal (11·1%). This is in line with the findings of a cross-sectional survey study which investigated the access to consumer technologies and willingness to use them to receive rehabilitation services among stroke survivors [36]. Their respondents' (*n* = 102) preference for in-person sessions and lack of interest for TR stems from concerns that TR would result in having fewer interactions with rehabilitation professionals (78·5%) and that quality of care over a virtual platform would be lesser as compared to in-person (71%) [36].

Our findings of the local preference for in person sessions may also be further substantiated by the relatively low interest to continue with TR (48·0%) in spite of the high satisfaction rates (84·0%). This may be attributed by how therapy sessions were delivered previously. All of the patients had prior experience of in-person rehabilitation and hence, are likely to have an understanding for rehabilitation to include physical touch. The patients may therefore be influenced to perceive remote delivery of rehabilitation to be lacking [27,36,37].

About one-fifth of the patients (21·0%) who declined TR expressed that it was due to lack of technology access or literacy at home. During CB period, family members were also restricted from visiting one another; patients who were less technologically-savvy thus faced difficulties in the set-up for TR. In addition, not completing and returning consent forms (67·9%) formed the top reason for patient dropouts. The primary mode to return completed consent forms was via electronic mail, of which some patients were not familiar with. This resulted in them not participating with TR. Lack of familiarity with technology forms a significant barrier in the adoption of TR at different points (i.e. submission of consent, receiving relevant information, set-up).

The observed lack of interest in this study, may be further attributed to a lack of exposure to TR locally [38]. Prior to the pandemic, there was low demand for remote care delivery, given the extensive transport infrastructure ensuring high accessibility within the island city state and access to home rehabilitation services.

### 4.1 Study limitations and recommendations

As the study was conducted retrospectively, we were not able to control the recruitment method to minimize loss of follow up from incomplete consent taking. The service was also implemented during an unsettling period. Training for use of TR devices and service was therefore conducted remotely which may limit control in the quality-of-service delivery.

Patients were unevenly distributed across the 12 disease classifications and allied health disciplines. As a result, it was difficult to draw correlation between the patient perception towards TR and their diagnostic groups.

In addition, participation in the patient experience survey was voluntary. This resulted in a low response rate which limits the statistical power in secondary analysis of patient's perception with the use of TR.

From our result, TR may be beneficial as an adjunctive tool in provision of patient education and advice tailored to their home environment. It may be used to facilitate for compliance and uptake to home exercise programme. For further implementations of the service, we would recommend closing the loop when seeking for consent, and to allow for more training of patients in the use of TR to increase familiarity of the service.

The roles and delivery of physiotherapy, occupational therapy, and speech therapy differ, and it may be meaningful to individually review patient response to the TR conducted by the different services. To have deeper understanding of the service, the future study could focus on specific diagnosis in order to draw meaningful relationship between the data in specific diagnosis group.

## 5 Conclusion

The pandemic had created a unique environment for us to study the local acceptability of TR. Only 24·2% of the eligible participants took up TR as a means to follow up with their rehabilitation during the CB period. Thus, suggesting that although TR can be viable tool of accessing healthcare during a pandemic, efforts to increase the ease and acceptance of this mode of care is necessary for its diffusion [38].

## Author note

This research has not been published or presented to any other parties.

This research did not receive any grant from any funding agency, commercial or not-for-profit sectors.

All authors do not have any conflict of interest.

## Declaration of Competing Interest

The authors declare that they have no known competing financial interests or personal relationships that could have appeared to influence the work reported in this paper.

## Figures and Tables

**Table:** 

**Primary condition for telerehabilitation**	**Criterion**
Orthopedics (post-operative/ musculoskeletal disorders)	**INCLUSION CRITERIA** • Post-operative cases • Follow-up appointment where manual therapy is not expected • Routine rehabilitation exercise program reviews as required • Education provision/consultation • Minimal to no radicular/radiculopathy symptoms • Single joint, mechanical pain **EXCLUSION CRITERIA** • Patient complains of severe pain • Complex pain presentation • Unable to set up and participate in tele-consultation
Neurology	**INCLUSION CRTIERIA** • Cerebrovascular accident (CVA) and traumatic brain injury (TBI) within 3 months with goal of functional return • Stable neurological • Medically stable • Minimal behavioral issues • Parkinson's Disease (Stage 2 on Hoehn and Yahr scale) • Able to follow 1–2 step verbal commands consistently with good attention span (at least 15 mins) • Acute nerve injuries with or without surgical repair **EXCLUSION CRITERIA** • Patients requiring more than 1 person, moderate assistance, for transfers • Patients who are bed bound or dependent in functional mobility
Vestibular	**INCLUSION CRITERIA** • Follow-up appointment where canalith repositioning maneuver is not • required • Exercise program review required (e.g. balance, gait, vestibulo-ocular reflex exercise) • Education provision for management of chronic conditions (e.g. Meniere's disease, vestibular neuropathy, chronic Benign paroxysmal positional vertigo) **EXCLUSION CRITERIA** • First episode of benign paroxysmal positional vertigo • Unresolved Benign paroxysmal positional vertigo since inpatient stay • Acute severe giddiness
Geriatric	**INCLUSION CRITERIA** • Patients who score 3–4 on the Clinical Frailty Scale • Diagnosed geriatric-related conditions • Stable post-operative review cases • Good cognition, able to follow 2–3 step instructions. • Reasonable attention span/ability to participate for >15 min • Patient will need a carer to be present during telerehabilitation if they score 5 and 6 on Clinical Frailty Scale and/or unable to follow >1 step command **EXCLUSION CRITERIA** Clinical Frailty Scale 7–9 to suggest caregiver training at inpatient wards, follow up with community team
Day Rehabilitation Centre	**INCLUSION CRITERIA:** • Able to follow 1 step command • If elderly or have a neurological background (non-independent) need care caregiver • If patients can be engaged virtually **EXCLUSION CRITERIA** • Must not have visual or hearing impairment
Clinical Exercise	**INCLUSION CRITERIA** • Clinical Frailty Scale 1–2 for chronic disease management and exercise consultation • Patients referred for chronic disease management through exercise • Medically stable
